# Essay on the Colic Produced by Lead

**Published:** 1838-01

**Authors:** 


### Art. IV.

*Essai sur la Colique de Plomb.* Par Augustin Grisolle, m.d., Membre de la Soc. Méd. d'Obser. de la Soc. Anatom., &c.—*Paris*, 1835. 4to. pp. 84.

*Essay on the Colic produced by Lead.* By Augustin Grisolle, m.d., &c. &c.

Few subjects connected with medicine have engrossed a larger share of the attention of medical men than the effects of lead on the animal economy. The physiologist, the toxicologist, the pathologist, have, in their respective walks, laboured in the solution of a variety of problems concerning them. The result is, that we possess much apparently valuable information on the phenomena which attend the introduction of lead and its compounds into the human body; and, what is most important, on the treatment of the morbid states to which it gives rise. But, on close enquiry into the character of the pathological facts, especially, which are looked on as established, we are forced to confess that vagueness and insufficiency of proof mainly distinguish them; and although there is, perhaps, no affection more completely under the control of the healing art than saturnine colic, such variety exists in the modes of treatment, and the alleged superiority of any mode above the rest is so little evident, that it must be admitted that we are, as regards that affection, far from possessing any thing like a perfect therapeutical code. The reason of this seeming wealth and real poverty is simple. Writers on this, as on a multitude of other medical questions, have contented themselves with recording a series of general conclusions founded on the hasty and insufficient observation of a few facts. The inductive method has been here on the same pretexts which have been hitherto so plausibly and too successfully urged against its use in other medical researches, utterly neglected. Yet it is impossible perhaps to find a disease so fitted in every point of view for its effective application as that which forms the subject of the treatise before us; for the objections most commonly urged against the value of the inductive and numerical methods, namely, the disparity of years, the difference of sex, of trade, of ordinary habits, existing between the individuals whose cases furnish general conclusions, in this instance scarcely exist. In a series of patients affected with saturnine colic, it will be generally found, that their trade is the same, their age almost constantly so, that they are with rare exceptions all males, their weekly gains exactly alike, and consequently their habits of living almost the same.

M. Grisolle, author of the work before us, has in it shewn that he fully understands the value of the numerical method. The importance of his results amply justifies his use of it, and is the best reward of his diligence. His position as house physician to the Hôpital Beaujon, where a great proportion of the cases of lead colic in Paris are treated, gave him opportunities which are rarely met with. The reader must not expect to find in this work a number of brilliant speculations, but what is infinitely more valuable, old opinions disproved or substantiated by the analysis of numerous and accurate facts, and some original propositions established in the same manner.

The author's results are obtained from fifty-eight cases of lead colic collected at the above hospital in 1834. The patients were chiefly supplied by the manufactories of ceruse at Pecq and Courbevoie.

In obedience to the precept of Cicero, " omnis institutio debet a definitione proficisci, ut intelligatur, quid sit id, de quo disputatur," our author commences by this concise and clear definition:

"The disease is characterized by violent abdominal pains, not increased by pressure, occurring in paroxysms, accompanied by bilious vomiting, obstinate constipation, slow pulse, and lastly by cramps and other painful sensations of the extremities." (P. 12.)

Passing to the causes of the affection M. Grisolle examines the influence of the seasons on its production. Having first ascertained from the heads of the establishments at Pecq and Courbevoie, that they had about the same number of workmen in employ at all periods of the year, he consults the hospital register, and finds that in the course of eight years 285 ceruse workers had been admitted. The facility of forming a correct diagnosis in cases of lead colic removes all fear of error in the register. These 285 cases occurred in the different months as follows:—

| | | | | | | | |
|---|---|---|---|---|---|---|---|
| January | 22. | April | 19. | July | 23. | October | 28. |
| February | 23. | May | 24. | August | 35. | November | 35. |
| March | 23. | June | 21. | September | 19. | December | 13. |

These numbers are extremely variable; and even by grouping the four hottest months, May, June, July, August; the four coldest, November, December, January, February; and the four of medium temperature that remain, the difference is trifling; the sums being 103, 93, and 89. Our author observes that " such difference is not considerable enough to allow us positively to affirm that season exercises a direct influence in the production of the affections resulting from the poison of lead," (p. 14.) The fact that the smallest and largest numbers are found in two adjoining winter months would, we think, warrant a more absolute assertion than this. The figures are of importance, inasmuch as they shew the general impression of the agency of season in causing the disease is erroneous; as also, to a certain degree the statement of Professor Chomel, who says (Dict. de Méd. 8, 383,) " lead colic is very rare at some periods of the year and becomes very frequent at others . . . especially in summer." This increase in the summer months is generally, he conceives, dependent on an increase in the activity of the manufactories at that period, and a consequent increase in the number of workmen employed and of hours devoted to labour daily. But M. Grisolle, as we have stated, ensured himself against this source of fallacy; his results therefore must be received as correct. The question is evidently important in a hygienic point of view.

Lead colic is liable to attack those who use the metal itself or any of its preparations: a variety of trades are consequently exposed to its ravages. But it would appear that some conditions of the metal are more deleterious than others, to a degree which could not be supposed à priori. Our author's researches shew that, while 285 workers of ceruse were treated at the Hôpital Beaujon in eight years, only thirty-two painters and colour-grinders received medical aid there during the same period. It must not be forgotten that the number of persons employed in the manufacture of carbonate of lead is much less considerable than that of the other classes just alluded to.

One half of M. Grisolle's fifty-eight patients were between thirty and forty years of age, but he justly remarks that before concluding hence that a particular age predisposes to the affection, it would be necessary to know the ages of all the workmen employed in the manufactories in 1834. He arrives at a result of some value by other means: "having arranged my patients in four sections, comprising those from æt. eighteen to thirty, thirty to forty, forty to fifty, and fifty to seventy, I found the mean length of their respective sojourns in the manufactories, sixty-five, sixty, fifty-eight, and thirty-seven days. Hence it appears that the number of days necessary to catch the disease diminished as the age of the subjects increased." (P. 15.)

Women are rarely employed in ceruse manufactories; hence colic is seldom observed amongst them. In the three cases which M. Grisolle met with, the affection was contracted in three months, whereas the mean stay of the males at Courbevoie was 101 days. Were these numbers on a larger scale, they would indicate a greater proneness in the female than in the male constitution to suffer from the noxious influence of lead.

Authors have laid down as an established fact that the manufacture of minium is more dangerous than of ceruse. The very contrary is shewn to be the truth by the calculations of our author: "eleven labourers were able to continue working at minium for seventy-three days without an attack of colic, while sixty-five days was the mean time that twelve others, employed on the carbonate, were able to continue their work," (p. 17.) M. Grisolle says nothing on the relative intensity of the colic when once declared. Our experience would lead us to consider that caused by minium as the most violent; we have not however tested the correctness of this opinion by the numerical method.

Foreign bodies are introduced into the economy in three ways: by cutaneous absorption, by absorption from the surface of the alimentary canal, and from that of the air passages. It becomes a question to determine by which of these ways lead penetrates into the interior of the body. M. Grisolle rejects the agency of the skin as wholly unproved. He founds his rejection on the following grounds: the frequent surgical use of preparations of lead, particularly the experience of Dupuytren who constantly employed the acetate as a local application to burns, and consequently to a denuded surface, and without the occurrence of any accident that could be ascribed to it. The instances quoted by Wall and Percival, in which colic and paralysis followed its external employment, appear to our author "unworthy of confidence on account of the total absence of detail in their relation. Instead of a complete description of the previous history and symptoms of the patients, I find only the opinion of the writer, of which it is impossible for me to ascertain the correctness," (p. 20.) But the facts recorded by Wall, if not accompanied with sufficient accuracy of detail to prove absorption through the skin, do not appear to us to be of a character to be thus set aside. The infant state of the physiology of cutaneous absorption leaves us without any à priori notions on the subject. We can scarcely doubt that, as M. Grisolle observes, if penetration do take place through the skin it must be a rare phenomenon in labourers, whose skin is rough, thick, and almost impermeable.

Our author considers the activity of pulmonary absorption in the introduction of lead established by numerous facts. We deem it unnecessary to enumerate them; the supervention of the disease in individuals who had simply slept in a newly painted room, (a case of the disease contracted under these circumstances was some time past observed by M. Louis,) is in itself sufficient to demonstrate the reality of such absorption.

M. Grisolle's cases prove in a remarkable manner that preparations of lead applied in a state of molecular division to the mucous membrane of the air passages, need not act as irritating bodies. "All my patients, with one exception, were totally free from cough, though working in an atmosphere thick with white lead; yet four of them had old thoracic affections. Three had been several years asthmatic from pulmonary emphysema, the fourth presented the symptoms of a collection of tubercles under the right clavicle," (p. 21.) Besides, not a single patient suffered from inflammation of the mucous membrane of the mouth and fauces, parts so evidently exposed to the constant action of the volatilized ceruse. Commenting on these facts, our author remarks, that daily observation proves the slight effect solid bodies reduced to an impalpable powder exercise on the mucous membrane of the bronchi. We must beg to offer our protest against this assertion. The researches of Drs. Hastings and Knight as clearly point out the destructive influence of volatilized mineral matter on the bronchial membrane, as they demonstrate that such influence does not induce tuberculization.* The number of cases alluded to by M. Grisolle is too small to warrant a general conclusion; or we might infer that lead in a pulverulent state is less deleterious as a bronchial irritant than other powders. In the celebrated case of the flint-cutters of Blois, Andral found that the molecules from the stone (to the inhalation of which the prevalence of pulmonary disorder among them was universally attributed,) did not and could not reach their mouths at all. Such an explanation of the harmlessness of the white lead cannot be admitted, for the whole atmosphere of the manufactories is charged with it in a pulverized state.

The effects of using water received in leaden cisterns in contact with the air, formerly a prolific source of colic at Amsterdam; the occurrence of saturnine colic from the use of wine adulterated with litharge, (a sort of epidemic of the affection has been more than once so produced at Paris); the occasional appearance of the disease in individuals taking preparations of the metal medicinally,—seem to M. Grisolle more powerful proofs of absorption by the digestive surface than can be furnished for either of the other modes. A question of practical interest springs from this part of the subject, viz. the quantity of lead which the economy can bear as a medicine. It unfortunately varies so much in different individuals that no general rule can be laid down. Thus M. Chomel has seen the exhibition of a drachm of liquid acetate of lead followed by colic; and a case has been given by M. Devergie in which such serious symptoms attended the ingestion of three pills of the same salt, that the attention of the magistrates was roused, and the apothecary who prepared the pills cited before them: on analysis, M. Devergie found a single grain only in

* Dr. Knight's case, in which the cicatrization of caverns took place while the patient was constantly exposed to the irritating influence of his trade, affords perhaps, as far as a single case goes, the most admirable refutation of the doctrine of irritation that the science possesses.

each of the remaining pills. On the other hand, Barbier (Mat. Méd. 3, 627,) has given 240 grains of the acetate in thirty-seven days, without any apparent ill effect on the digestive and nervous systems. M. Fouquier has given eighteen grains per diem for three months without intermission with similar freedom from accidents. We have no doubt but that some common character exists in the different series of patients on whom the action of the medicine is so widely different. This common character can only be discovered by accurate observation and the numerical method.

We gave some important observations on this subject, in our last volume, by Professor Mitscherlich. (See Selections from the Foreign Journals, p. 208.)

The presence of the acetate in the blood has been ascertained by Tiedemann. He detected it in the mesenteric and splenic veins of dogs to whom he had administered that salt. Hence, say the analogical reasoners, we must find evidence of the presence of lead in the blood and secretions of individuals affected with lead colic. Yet M. Grisolle sought in vain for any traces of its existence in the urine, in the matter vomited, in the blood, saliva, peritoneal serosity, bronchial mucus, and feces of his patients. The experiments were made with sulphureous preparations. The same is not the case with ferrugineous compounds: iron has been detected in the urine in the state of prussiate by Mojon of Genoa.

M. Grisolle extended his researches on the effects of lead to the inferior animals. He found them very different in different species. Thus, at one manufactory he saw horses that had been employed for ten and fourteen years, and which were covered with a coating of white lead, and yet enjoying perfect health. Cats bring forth their young in a bed of ceruse, and are perfectly exempt from its ill effects; yet cats and dogs die, almost without an exception, from frequenting the establishments.

On the general action of lead before the supervention of colic, and the precursory symptoms of the latter, our author has given two chapters: for these we must refer our readers to the original.

All the symptoms of the affection, and their progress, are placed before us with clearness and accuracy; each symptom is then examined apart in all its details. We shall content ourselves with noticing a few points among these.

It is matter of common belief that the abdominal pain in lead colic may *always* be relieved by pressure. Such is not the truth, however. In forty out of fifty-two cases, the pain was assuaged by gradual and continued pressure; in seven it was uninfluenced by it, and in the remaining five augmented. We have ourselves observed cases of the latter peculiarity at Beaujon: it appeared to us dependent on an inflammatory complication. Retraction of the abdominal parietes (another symptom announced as constant in its occurrence, by some authors,) was only observed fifteen times in forty-six cases; in the remaining thirty-one patients, it had its ordinary degree of development. The fact is, that not unfrequently the size of the abdomen is considerably increased.

The appearance of the tongue (a point of so much importance, according to the doctrines of the physiological school, and which the accurate M. Louis has shewn to be of infinitely less importance than that school would have it,) was not neglected by our author. He found it humid and of natural colour, and slightly furred in half his cases. In one instance during the treatment, the tongue became greyish, furrowed, and dry; the pulse rose to eighty-eight, with febrile heat. These symptoms lasted only twenty-four hours, when the tongue recovered its moisture. These results coincide exactly with those obtained by M. Louis in the same affection. (*Fièv. typh.* 2, 103.)

According to the assertion of Stoll, patients who vomit during the colic recover more easily and rapidly than those who do not present this symptom, though the sufferings of the latter are less acute. The result of M. Grisolle's observation is completely at variance with the opinion of the celebrated German. Thus, the mean duration of the affection in patients who vomited was eight and a quarter days; in those who had slight nausea and bitter taste in the mouth, five days; in those who were without gastric symptoms, four and a quarter days. The experience of M. Grisolle is again in contradiction with that of Stoll on the character of the pulse. The latter states that its hardness is uniformly greater than in any other affection: M. Grisolle, having attentively sought this peculiarity, found it in three cases only.

The modifications of the urine were slight. They were confined to trifling decrease in quantity, red colour, and deposit of sediment. Its excretion was seldom attended with scalding. M. Grisolle never observed a sudden stoppage of the stream of urine; a phenomenon stated to occur sometimes, by Stoll, Bauer, and Dance, and attributed to sudden contraction of the urethra.

We are next presented with an elaborate examination of the various lesions of sensibility noted in the progress of the disease. These were by no means limited to the primitive seat of the affection, the abdomen. Pain was the most remarkable of them, and is treated of in so many separate sections, as it occurred in the genitals, extremities, loins, thorax, and head. That in the genitals (usually supposed to exist in the testicle,) was, in the majority of cases, placed in the spermatic cord. It occurred in the fourth part of the patients, and was in many instances attended with retraction of the testis, but never with redness or tumefaction of that organ. The character of the lumbar pain seemed favorable to the theory of Astruc, as to the seat of the disease being in the medulla spinalis. It was increased by pressure, and, radiating from the spine, passed round to the epigastrium, sternum, umbilicus, and walls of the pelvis, exactly as that symptomatic of a lesion of the medulla spinalis.

Many authors have asserted that the pain in lead colic is subject to nocturnal exacerbation. Stoll goes so far as to consider this peculiarity more marked even than in syphilis. On this M. Grisolle remarks, "I am inclined to think this an exaggerated statement; for, of eighteen patients carefully questioned on this point, ten only suffered an exacerbation of pain by night." (P. 44.) We regret that we have not room for more detailed notice of this part of the treatise.

The lesions of innervation observed by M. Grisolle were not confined to these. The most important affections were epilepsy, paralysis with coma, and general trembling. The intellectual faculties were more or less disordered in two cases: in one there was a state bordering on dementia, in the other the symptoms resembled those of delirium tremens. Epilepsy occurred twice only. In both cases it came on suddenly, and the proper symptoms of colic were very slight: both patients died within thirty-six hours from the moment of the attack. In these instances the author does not attribute the convulsions "to sympathy, nor to a reaction of the nervous centres produced by the intensity of the pain, but considers it necessary to admit an immediate action of the lead on the cerebro-spinal system." (P. 46.) We are fully disposed to agree with M. Grisolle in his mode of explaining these nervous phenomena. It seems, indeed, impossible to suppose them, as some authors have done, a sympathetic effect of the abdominal affection, when we recollect that they supervene occasionally before the colic itself, or persist frequently after its total cessation.

The nature of lead colic is a point which has given rise to much warm dispute. Four different opinions have found abundance of supporters. In giving a sketch of them, we shall endeavour to settle, at the same time, the question of the "anatomical character" of the disease.

1st. The disciples of Broussais have, of course, not failed to discover in the few cases where the use of the scalpel was possible, the omnipresent gastro-enteritis; broadly asserting (*Ann. de Méd. Phys.* 9.) that, in every instance in which examination of individuals affected with lead colic had been made, the mucous membrane of the intestinal canal was found inflamed. Yet not a single author, unprejudiced by systems, has found the least sign of inflammation in the part to which we have alluded. Our author's cases add to the number already laid before the profession by Andral, Louis, Laennec, Martinet, Rufz, &c., in which the most accurate search failed to detect inflammatory changes. In M. Grisolle's two cases, there was neither "anormal coloration, softening, or thickening of the tissues composing the digestive tube," (p. 52;) although in these cases there was an additional cause, according to the Broussaian theory, for the production of enteritis,—the use of drastic purgatives. Death had occurred in the two cases from epilepsy; a third case, of fatal termination from cholera, was, of course, unfit to aid in solving the question. "If," observes M. Grisolle, "lead, in the molecular state, produced even slight inflammation by contact with the digestive mucous membrane, it appears to me that the symptoms of colic should be more rapid in their occurrence and intense in their degree, when the metal is introduced directly into the alimentary canal. Now, the exact contrary is the truth: those who respire emanations from lead contract the colic more easily than those who swallow the metal; which, too, when given in large doses, induces the symptoms, not of saturnine colic, but of poisoning; that is, of a violent inflammation of the mucous membrane of the digestive tube." (P. 52.)

2dly. Coarctation of the intestine has been laid down by some writers as the efficient cause of lead colic. Our author justly rejects this, as constituting a peculiar character of the disease. Irregular contraction of the intestine is a phenomenon observed after death from a variety of affections, and cannot be considered otherwise than as an accidental circumstance in colitis pictonum, when it occurs. In six cases examined by Andral, there was no diminution of the caliber of the intestine. We must not omit that, in one of four cases in which M. Grisolle attempted to introduce an œsophagus tube into the colon per anum, he was able to pass only eight inches; but it is impossible to assert that its stoppage was not due to a collection of hardened fæces. In the other cases it penetrated to the end, and without the least difficulty.

3dly. The opinion which places the seat of lead colic in the medulla is supported by hypothesis alone. In one of his epileptic cases, M. Grisolle found a general diminution of consistence in the medulla; the brain was similarly affected in both subjects. But these cases can in no wise throw a light on the pathological anatomy of the colic itself. They corroborate, as far as regards saturnine epilepsy, the details of cerebral lesion given by Miguel, Laennec, and Caseaux. But, in a very large majority of cases of epilepsy due to the poison of lead, no such lesions have been found.

4thly. The opinion that the disease is of a purely nervous nature is adopted by M. Grisolle. "The absence of fever, the violence of the pain, its relief by pressure, and special character of returning by paroxysms; its occasional radiation in the known course of certain nerves; the white flat tongue, obstinate constipation, scalding urine; the interruption of the symptoms, which sometimes lasts for several days, suffice to prove its nervous nature." (P. 54.) This opinion is borne out by "the total absence of any appreciable lesion in the intestinal canal, and the almost constantly happy effects of narcotics and irritants." (P. 55.) M. Grisolle does not attempt to decide whether the disease be seated in the filaments of the cerebral nerves or of the sympathetic. "However this may be," he adds, "the spinal marrow would appear, in severe cases, to share the poisonous influence of the lead, whether the metal exercises a direct modification on it, or through the extensive communications which exist between the ganglia and rachidian nerves."

M. Grisolle presents us with nothing new on the prognosis. An interesting calculation is given by Professor Bouillaud, from which it appears that, in 3,569 cases of lead-colic, death occurred *ninety-five* times, or a little less than once in thirty cases. All these deaths were the effect, not of the colic itself, but of various complications, particularly affecting the nervous system. The patients who furnished materials for the above calculation were, almost without exception, treated at La Charité.

The treatment of the affection is subdivided by the author into prophylactic and curative. The use of certain hygienic precautions in the mode of construction of the manufactories, and the conduct of the workmen, constitute, according to M. Grisolle, the only true prophylaxis of lead colic. In this he is supported not only by the favorable results obtained from such precautions, but by the complete inefficacy of certain medications, the prophylactic virtues of which have been extolled from time to time. The essential conditions for attaining the greatest possible healthiness in the establishments is, that they should be built on an elevated situation or on the banks of a river; that there should be a continual current of air through them; and that the necessary machines be covered as much as possible, so as to prevent the dust from reaching the workman.

With respect to the labourers themselves, the evil influence of spirituous excess points out the necessity of abstinence: the day's work should never be commenced on an empty stomach; for it appears that those whose habit it was to do so "were seized with colic after *forty-eight* days' work, on an average; while those who followed the contrary plan remained unaffected *sixty-five* days." (P. 63.) The presence of white or red lead in the rugæ and depressions of the skin indicates the necessity of using the warm bath occasionally. We may remark, that M. Gendrin has somewhere attributed to the oxide and carbonate of lead, thus combined with the epidermis, the various effects of the metal distinct from the abdominal affection. Unquestionably, as M. Grisolle believes, such an opinion must be erroneous; "not that we are authorized to deny cutaneous absorption, but because the presence of molecules of lead on the skin is a *constant* phenomenon, in my opinion, inasmuch as I never looked for it without succeeding in my search." (P. 64.) Our experience coincides with that of M. Grisolle on this point: in upwards of *thirty* cases, we have invariably found the metallic incrustation simply with the naked eye, and without the help of any chemical reagent. In order effectually to remove it, M. Grisolle recommends the use of the sulphur bath, followed by a simple warm bath, to detach the recently formed sulphuret.

Our readers are probably aware that in 1832, M. Gendrin, to whom we have just alluded, presented a memoir to the Institute, undertaking to prove the preventive powers of sulphuric acid against the development of lead colic. The results obtained by our author on this question (who has put to the test the accuracy of M. Gendrin's allegation,) are as follows:*

"Nineteen labourers, who had used the sulphuric tisane, were obliged to give up their work, after an average stay of fifty days; whereas, twenty-five others, who employed no prophylactic measures, did not contract the disease until after ninety days." (P. 65.)

These calculations are made from all the patients, taken without distinction; no matter from what manufactory they came.

"Next comparing amongst themselves the labourers of the same establishment, I found, for that of Pecq, that those who drank no sulphuric acid fell ill after forty-four days, while those who made use of it resisted forty-nine days. At Clichy, seventy-six days was the mean stay of those who used none of the tisane, fifty that of those who employed the prophylactic treatment of M. Gendrin." (P. 65.)

From these facts our author deduces the uselessness of sulphuric acid as a prophylactic. Our limited experience in this matter accords with his. We have observed in the few cases where the workmen had been prevailed on to use enough to give it a fair trial, that it was wholly wanting in the miraculous virtues attributed to it by M. Gendrin. It has even produced slight diarrhœa, loss of appetite, and gastric derangement in many cases after a few days' use. If the testimony of four cases may be relied on, nitric acid is equally powerless in fulfilling the same indication; our author's experience of it does not extend further.

We have already alluded to the multiplicity of modes of treatment of lead colic that claim for themselves the first rank in point of efficiency. The strong desire to be useful has in some cases blinded medical innovators as to the value of their favorite treatment; in others attachment to particular systems has been the cause of error. Nothing is more easy

* The sulphuric tisane was composed of a drachm and a half of sulphuric acid at 66° to three litres of water, and several ounces of moist sugar. It was supplied *ad libitum* to the workmen.

than to persuade oneself of the superior powers of any given treatment. Cases of mild and severe character, those treated at their outset or at their decline, need only to be jumbled together, and favorable results must necessarily be obtained. But those who seek truth, and not specifics, avoid this unfair method of proceeding. A single case will illustrate our assertion. Our readers, no doubt, recollect the praises bestowed on the alkaline principle of the willow, salicine: it was announced by more than one as a sovereign remedy for intermittents, and, from its cheapness, recommended to our sympathies as the poor man's cure. Yet, when tried by an accurate observer, its reputation was proved to be wholly unmerited. Professor Chomel (the observer to whom we refer,) made his experiments on those patients only whose affection was not fast tending to a natural recovery, and in whom it was not of that mild character which allows of a favorable termination without any treatment at all.

The method of treatment most frequently employed in the cases of M. Grisolle, whose details on this part of the subject exhibits the most praiseworthy accuracy, was that by purgatives and opiates combined. We shall do our author the justice to give, in his own words, the summary of his observations.

"The following enema was administered, either alone or along with opium, to sixteen of my patients:—Dec. Sennæ, ℥j.; Sodæ Sulph., Mel. Mercurial. Ann. āā ℥ij.; Aquæ, q.s. Its exhibition was uniformly followed, within from thr ee to twelve hours, by a remarkable degree of relief. Of these sixteen patients, seven were affected with violent colic; altogether each of them took during the treatment, which lasted, on an average, five days and a quarter, six enemata and five grains of opium. I dated the recovery from the moment the abdominal pains ceased, the alvine evacuations were restored, the appetite returned, and digestion was easy. The other nine individuals were affected with less severe colic than the preceding; they recovered, on an average, in four days and a quarter, after having had four purgative enemata and three grains of opium. In half the cases just analysed, I noted vomiting; but this symptom ceased on the supervention of the alvine evacuations. The cessation of pain must not be ascribed to the opium alone; for it was not administered to six of the above patients, and in every case it was not given until the constipation was overcome, and considerable amelioration was obtained in other respects. When the colic was of the severe kind, the first enema rarely produced much effect. It was occasionally necessary to administer several, from three to five, for example, one after another, to overcome the constipation when very obstinate. The fæces first voided were either dry and black, or yellow and of slight consistence." . . . "Six patients were treated at the same time with purgatives by the mouth and in enemata. This last method produced the most abundant evacuations and rapid relief. Recovery took place in this series in seven days and a quarter. The purgatives used were three in number: Castor-oil, ordinarily combined with croton-oil, in the dose of from one to three drops. The latter never produced superpurgation; sometimes, indeed, it had no effect at all. The 'huile d'épurge' was also administered in drachm doses, and almost always produced vomiting, with or without purging." (P. 69.)

"It became necessary to give up the use of purgatives in four cases, from theirwant of success, and on account of the supervention of febrile symptoms. Venesection was performed in two of these cases. The blood was very rich in both instances, and buffed in one of them. The serosity, small in quantity, was treated by the reagents of the salts of lead, without presenting either black coloration or precipitate." (P. 71.)

M. Grisolle's experience is unfavorable to the use of opiates alone, as recommended by Stoll; and to the modes of treatment by tobacco, sulphuric acid, and antiphlogistics: on the latter, particularly, he has given some very interesting information.

